# Association Medical Officers of Late Confederate Army and Navy

**Published:** 1874-06

**Authors:** 


### CONVENTION OF EX-CONFEDERATE SURGEONS.

ATLANTA, GA., May 20, 1874.

The convention assembled in the Senate Chamber at 11 A. M.

On motion, Dr. S. H. Stout was called to the chair, and Dr. Charles Pinckney requested to act as Secretary. On taking his seat Dr. Stout returned his thanks, and made appropriate remarks touching the object of the assemblage of medical officers of the late Confederate States army, and the ends which it is proposed to accomplish, and paid a feeling, but just, tribute to the heroism and self-sacrifice in the line of duty, which he had observed in many of the medical officers who had been under his supervision during the great struggle.

Upon motion of Dr. P. A. Holt, of Florida, it was resolved that the medical officers present record their names, with date of commission, capacity in which they served, and date of discharge or parole.

On motion of Dr. Goldsmith, the following committee was appointed by the chair to make nominations for permanent officers of the body, namely: Drs. W. T. Goldsmith, of Georgia; P. A. Holt, of Florida; W. A. Carswell, of Georgia; E. D. Newton, of Georgia; W. A. Greene, of Georgia; J. W. Oslin, of Georgia; H. F. Campbell, of Georgia; A. G. Emery, of Alabama; M. W. Francis, of Alabama.

On motion, Drs. John M. Johnson, formerly of Kentucky; S. H. Stout, formerly of Tennessee; A. C. Fox, formerly of Virginia; and E. J. Roach, formerly of Maryland, were added to the committee.

A letter was presented by Dr. W. F. Westmoreland, of Georgia, and read by the Secretary, from ex-Surgeon General S. P. Moore, of Virginia, expressing his regret that he could not be present, and expressing his warm approval of, and hearty co-operation in, the objects of the meeting, and desiring the enrollment of his name as a member.

On motion of Dr. Logan, of Georgia, the following committee was appointed by the chair to report business for the action of the convention, namely: Dr. J. P. Logan, H. F. Campbell, Robert Battey, E. J. Roach and W. O. Owen.

Upon motion of Dr. Miller, of Georgia, a recess was taken until three o'clock P. M., to give the committees time to report.

AFTERNOON SESSION.

Dr. S. H. Stout in the chair.

Dr. Goldsmith submitted the following:

"The committee on permanent organization of the convention of medical officers of the late Confederate States Army and Navy, beg leave to make the following report:

"We respectfully nominate late Surgeon General, Dr. S. P. Moore, of Richmond, to be permanent President of this convention, and Dr. Henry F. Campbell of Georgia, to be Vice-President at large, also, District Vice-Presidents: Dr. James E. Claggett, of Maryland; Dr. Hunter McGuire, of Virginia; Dr. S. S. Stachwell, of North Carolina; Dr. A. N. Talley, of South Carolina; Dr. W. F. Westmoreland, of Georgia; Dr. P. A. Holt, of Florida; Dr. C. J. Clark, of Alabama; Dr. S. V. D. Hill, of Mississippi; Dr. E. S. Drew, of Louisiana; Dr. J. N. Hayden, of Texas; Dr. Paul F. Eve, of Tennessee; Dr. D. A. Linthicum, of Arkansas; Dr. D. W. Yandell, of Kentucky; and Dr. L. T. Pim, of Missouri.

"The committee also nominate as Vice-Presidents at large all former medical officers of the U. S. Army and Navy who resigned their positions for service in the Confederate States Army and Navy. They also recommend that the medical officers of the late C. S. Navy be requested to co-operate with the officers of this convention. They also nominate Dr. S. H. Stout for Secretary; Dr. Charles Pinckney for Assistant Secretary; and Dr. Edwin D. Newton for Treasurer of this convention."

On motion of Dr. W. F. Westmoreland, it was *resolved*, that all surgeons, assistant surgeons, and acting assistant surgeons of the late C. S. Army and Navy be admitted to register as members of the convention.

A telegram was read from Vicksburgh, Mississippi: "The undersigned, ex-Confederate medical officers, send greeting; they regret their inability to be with you in person, and assure you of their sympathy and co-operation in this beginning of annual reunions. (Signed) D. W. Booth, L. M. Capers, J. R. Hicks, J. R. Barnett, J. M. Hunt, P. O'Leary, W. E. Brickell, G. W. Howard and P. F. Whitehead.

The committee on business offered the following suggestions in their report, which were amended and adopted, viz:

"1st. We recommend that this organization shall be called the 'Association of Medical Officers of the late Confederate Army and Navy.'

"The objects of the Association shall be the collection and preservation of the medical records and statistics of the late C. S. Army and Navy; the collection and publication of such scientific facts as may be useful to the Association and to the medical profession; the preparation and publication of biographical notices of deceased members of the late medical staff, and the cultivation of social and friendly intercourse amongst its members.

"3d. The officers shall be a President, a Vice-President at large, one Vice-President from each State represented in the said army, a Secretary and Assistant Secretary, and a Treasurer, who shall be elected annually.

"4th. The meetings shall take place annually, at such time and place as the Association may determine.

"5th. There shall be an assessment to meet current expenses, of such sum as may be determined by the body.

"6th. There shall be the following standing committees, viz: Committee on Hospital Service, Committee on Field Service, Committee on Necrology, Committee on Miscellaneous Reports, and Committee on Hygiene. There shall also be a special committee, consisting of three members and the Secretary, invested with discretionary powers, of which the Secretary shall be chairman, to whom shall be referred all matters for publication.

On motion of Dr. Battey, it was *resolved*, that a committee of five be appointed to prepare and submit to the next meeting of the Association a form of permanent constitution and by-laws for its government. The chair appointed Drs. Battey, Cumming, Logan, Miller and Newton.

SECOND DAY.

Dr. H. F. Campbell, Vice-President, in the chair.

A telegram was read from Dr. Hunter McGuire, of Richmond, Virginia.

Dr. Logan presented a communication from Dr. John M. Johnson, President of the Savannah Association of Medical Officers of the late Confederate States Army and Navy.

On motion of Dr. Stout, it was *resolved*, that a committee of five members be appointed to prepare an address to the members of the medical staff of the late Confederate Army and Navy, explaining the objects of this Association, and asking their co-operation.

On motion of Dr. Logan, it was *resolved*, that we recommend the formation of local societies for the furtherance of the objects contemplated by this Association.

Dr. Logan read a telegram from Dr. D. W. Yandell, of Kentucky, expressing his hearty co-operation and bidding the Association God-speed.

Dr. Westmoreland had letters from Dr. J. T. Gilmore, of Mobile, and Dr. C. B. Leitner, of Geneva, Georgia, regretting their inability to be present, and wishing the organization every success.

On motion of Dr. Andrews, it was *resolved*, that the newspapers throughout the late Confederate States be requested to publish the address to the Medical Officers of the late C. S. Army and Navy, which will be issued by the committee appointed for that purpose.

On motion of Dr. W. F. Westmoreland, it was *resolved*, that a committ of three be appointed to confer with the Surgeon General U. S. A. in reference to the captured archives of the Medical Department of the late Confederate States, as to the extent to which they can be used by this body, and all other matters pertaining to the publication of the same, and report the result of the conference at the next meeting of this body.

On motion of Dr. Goldsmith, it was *resolved*, that all ex-Confederate medical officers be requested to furnish to Dr. S. H. Stout, Secretary, Atlanta, Georgia, their names, with their rank, and period of service, etc., for enrollment as members of this association, with the understanding that they shall be considered members upon such enrollment.

Dr. Stout read a communication tendering to the Association the records of the Hospital Department of the Army of Tennessee in trust, provided that a safe place be secured for their deposit. (Signed) S. H. Stout, Medical Director for Hospitals of the late Confederate Army of Tennessee. The communication was received and ordered to be recorded.

Dr. Logan read the following: The Confederate medical records belonging to the late Dr. A. J. Foard, Medical Director of the Western Army, now in my possession and at my disposal, are hereby tendered to this Association, upon condition that proper steps are taken for their preservation and utilization by this body. (Signed) J. P. Logan, M.D.

Dr. Newton stated that Governor Smith had kindly tendered the use of a room in the capitol building at Atlanta for the deposit of archives.

On motion of Dr. Crawford, it was *resolved*, that the tender of valuable records and papers by Drs. Stout and Logan be accepted, and the thanks of the Association be returned to these gentlemen.

On motion of Dr. Green, *resolved*, that former medical officers of the U. S. Army and Navy, who resigned their positions to take service under the Confederate States, be registered, upon their application for membership, as honorary Vice-Presidents of this body.

On motion of Dr. Newton, *resolved*, that the Vice-Presidents of each State be authorized to appoint an executive committee to assist in procuring reports, organizing auxiliary societies, and promoting the objects of this Association.

AFTERNOON SESSION.

On motion of Dr. Stout, *resolved*, that the Committee on Constitution and By-Laws be instructed to take into consideration the feasibility of so organizing the Association as a whole, and in its various divisions, as to preserve the *esprit du corps* of each branch of the service, and of each department thereof; and of so arranging the work of the Association, and of local organizations co-operating with it, that the labor and contributions of officers of one military department shall not be confused with those of another; the infantry and artillery with the cavalry arm; those in a given brigade, division or corps with those in another; the hospital service with the field service; nor the work of the jurisdiction of one medical directorship with that of another. And further, that it is the sense of this Association that the records of the Army and Navy Medical Examining Boards should be presented and their archives preserved.

*Resolved*, That Dr. J. D. N. Cullen, Medical Director of Longstreet's corps, be requested to prepare a sketch of the organization and service of the Medical Department of the Army of Northern Virginia; to be delivered at the next meeting.

*Resolved*, That Dr. C. A. Carrington, Medical Director of the hospitals at Richmond, be requested to prepare a similar paper, to be delivered at the same time.

*Resolved*, That Dr. E. A. Flewellen be requested to prepare a similar paper on the field service of the Western Army; and that Dr. S. H. Stout be requested to prepare one on the organization of the Hospital Department of the Army of Tennessee; also that Dr. A. N. Talley be requested to prepare a report of the Army Medical Examining Board.

On invitation, the Vice-President, Dr. Campbell, addressed the body upon some of his observations in the Army of Virginia. He alluded to the successful treatment of intermittent fever by the use of oil of turpentine, the scarcity of quinine necessitating search for substitutes for it. He thought the turpentine increased the renal secretion, and eliminated thereby the materies morbi. This, and other expedients of necessity, should be brought out and put upon record. He related a case of wound in the calf of the leg, ball passing between the tibia and fibula. There was great inflammation threatening gangrene, discharge of dirty sanies, and hemorrhage from the posterior tibial artery. The question of amputation or ligation of the artery was discussed. It was decided to ligate the femoral in scarpa's triangle to both control the hemorrhage and at the same time modify the inflammation. The operation was successful, and a good limb was saved. He related several other cases of ligation of principal arteries for the purpose of modifying inflammation, with good result. He then discussed elaborately the subject of inflammation, its causes, characteristics, and the methods of treatment.

*Resolved,* That an abstract of Dr. Campbell's remarks upon inflammation and its treatment be furnished the Secretary for publication; and that Dr. C. be requested to further elaborate his ideas upon the subject in an essay to be read at the next meeting of the Association.

*Resolved,* That the Committee on Hospital Service be instructed to obtain information in regard to the results of the use of Dr. Nathan R. Smith's anterior splint in fractures of the lower extremity in Confederate hospitals.

The President appointed the following chairmen of committees, who are to be allowed to select their own assistants, namely: Committee on Address, Dr. S. H. Stout, Atlanta, Ga.; Committee on Hospital Service, Dr. J. B. McCaw, Richmond, Va.; Committee on Field Service, Dr. D. W. Yandell, Louisville, Ky.; Committee on Naval Service, Dr. W. H. Spotswood, Pensacola, Fla.; Committee on Necrology, Dr. J. P. Logan, Atlanta, Ga.; Committee on Miscellaneous Reports, Dr. E. D. Newton, Athens, Ga.; Committee on Hygiene, Dr. W. H. Cumming, Atlanta, Ga.; Committee on Membership, Dr. J. D. N. Cullen.

Dr. Newton read a memoir of Medical Director LaFayette Guild, in which he paid a merited tribute to the official and private character of the deceased. Referred to Committee on Necrology.

*Resolved*, That Drs. Eldridge, Stout and Barnes be appointed a committee to collect the facts in regard to the medical and sanitary history of Andersonville prison.

NIGHT SESSION.

Dr. Logan suggested the death of Dr. Carrington, and moved that Dr. Charles H. Smith be appointed to prepare the proposed essay in his place. Adopted.

*Resolved*, That Dr. W. H. Cumming be requested to prepare a paper on vaccination, its results, normal and abnormal, as manifested in the army of the Confederate States, to be presented at the next meeting.

*Resolved*, That the Committees on Field Service and Hospital Service be instructed to report upon the results of the use of indiginous remedies in the late Confederate States, at the next meeting.

After the usual resolutions of thanks, the Association adjourned to meet in Richmond, Virginia, on the first Wednesday in July, 1875.

